# Ageing increases reliance on sensorimotor prediction through structural and functional differences in frontostriatal circuits

**DOI:** 10.1038/ncomms13034

**Published:** 2016-10-03

**Authors:** Noham Wolpe, James N. Ingram, Kamen A. Tsvetanov, Linda Geerligs, Rogier A. Kievit, Richard N. Henson, Daniel M. Wolpert, Cam-CAN, Lorraine K. Tyler, Carol Brayne, Edward Bullmore, Andrew Calder, Rhodri Cusack, Tim Dalgleish, John Duncan, Fiona E. Matthews, William Marslen-Wilson, Meredith A. Shafto, Karen Campbell, Teresa Cheung, Simon Davis, Anna McCarrey, Abdur Mustafa, Darren Price, David Samu, Jason R. Taylor, Matthias Treder, Janna van Belle, Nitin Williams, Lauren Bates, Tina Emery, Sharon Erzinçlioglu, Andrew Gadie, Sofia Gerbase, Stanimira Georgieva, Claire Hanley, Beth Parkin, David Troy, Tibor Auer, Marta Correia, Lu Gao, Emma Green, Rafael Henriques, Jodie Allen, Gillian Amery, Liana Amunts, Anne Barcroft, Amanda Castle, Cheryl Dias, Jonathan Dowrick, Melissa Fair, Hayley Fisher, Anna Goulding, Adarsh Grewal, Geoff Hale, Andrew Hilton, Frances Johnson, Patricia Johnston, Thea Kavanagh-Williamson, Magdalena Kwasniewska, Alison McMinn, Kim Norman, Jessica Penrose, Fiona Roby, Diane Rowland, John Sargeant, Maggie Squire, Beth Stevens, Aldabra Stoddart, Cheryl Stone, Tracy Thompson, Ozlem Yazlik, Dan Barnes, Marie Dixon, Jaya Hillman, Joanne Mitchell, Laura Villis, James B. Rowe

**Affiliations:** 1 Department of Clinical Neurosciences, University of Cambridge, Cambridge CB2 0SZ, UK; 2 Medical Research Council Cognition and Brain Sciences Unit, Cambridge CB2 7EF, UK; 3 Computational and Biological Learning Laboratory, Department of Engineering, University of Cambridge, Cambridge CB2 1PZ, UK; 4 Centre for Speech, Language and the Brain, Department of Psychology, University of Cambridge, Cambridge CB2 3EB, UK; 5 Behavioural and Clinical Neuroscience Institute, University of Cambridge, Cambridge CB2 3EB, UK; 6 Cambridge Centre for Ageing and Neuroscience (Cam-CAN), University of Cambridge and MRC Cognition and Brain Sciences Unit, Cambridge CB2 7EF, UK

## Abstract

The control of voluntary movement changes markedly with age. A critical component of motor control is the integration of sensory information with predictions of the consequences of action, arising from internal models of movement. This leads to sensorimotor attenuation—a reduction in the perceived intensity of sensations from self-generated compared with external actions. Here we show that sensorimotor attenuation occurs in 98% of adults in a population-based cohort (*n*=325; 18–88 years; the Cambridge Centre for Ageing and Neuroscience). Importantly, attenuation increases with age, in proportion to reduced sensory sensitivity. This effect is associated with differences in the structure and functional connectivity of the pre-supplementary motor area (pre-SMA), assessed with magnetic resonance imaging. The results suggest that ageing alters the balance between the sensorium and predictive models, mediated by the pre-SMA and its connectivity in frontostriatal circuits. This shift may contribute to the motor and cognitive changes observed with age.

## Abstract (web summary)

Decline in sensorimotor skills with age may be due to an overreliance on the prediction of the sensory consequences of one's actions. Here the authors show that sensorimotor attenuation increases with age, and that this is associated with structural and functional changes in frontostriatal circuits.

## Graphical abstract

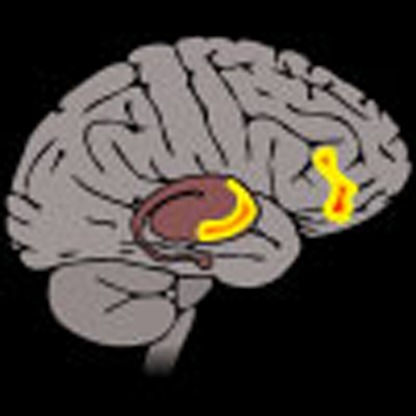

Healthy ageing is associated with widespread brain changes^1^, including the sensory and motor systems. Such changes lead to a deterioration in the control of movement. Many impairments in basic and skilled movements have been observed with ageing, including balance^2^, speed^3^ and motor learning^4^. Together, these put older people at greater risk, for example, of falls^5^, which increase the overall disease burden^6^.

From reaching and grasping to gait and balance, motor skills require an accurate integration of sensory and motor signals to estimate the state of the body. To achieve this, afferent information from peripheral sensors is combined with predictive signals generated by internal forward models of movement^7^. Using forward models, the brain predicts the sensory consequence of one's action, making the system robust in the face of delays or noise associated with sensory processing^8^ and improving performance in uncertain environments where sensory noise is increased^9^,^10^.

Noise in this context means the corruption of sensory signals by irregular fluctuations that interfere with accurate information transfer. Such interference can originate at the level of the peripheral sensory receptors as well as the peripheral and central neurons^11^. Reducing the impact of noise in the sensorimotor system can be achieved by Bayesian integration, using a weighted average of sensory evidence and a ‘prior' prediction. The weighting of these signals is dependent on their relative precision (the inverse of noise). Changes in the precision of sensory information relative to predictive forward models would therefore have a widespread impact on motor control^12^. However, it is currently unclear how age affects sensorimotor integration.

The integration of sensory information with predictive signals from forward models can lead to sensorimotor attenuation^13^, in which the sensory consequences of a voluntary action are perceived as weaker than the same sensory event when it is externally generated^14^. Attenuation is proposed to be mediated through an ‘efference copy' of the motor command, which is used to predict the consequences of movement^15^. Precise prediction is in turn used to attenuate the perception of the consequences of one's own action, facilitating the distinction between self- and externally caused events and enhancing the salience of environmental stimuli^14^,^16^. Conversely, imprecise prediction with greater weighting of sensory signals and reduced sensorimotor attenuation has been suggested to lead to abnormalities in the attribution of action in neuropsychiatric disorders, such as schizophrenia^16^ and psychogenic movement disorders^17^.

Attenuation of the sensory effect of one's action has been reported for somatosensory^14^, visual^18^ and auditory^19^ perception. Its exact neural mechanism is not entirely clear, but functional neuroimaging studies have suggested that sensorimotor attenuation is associated with both subcortical regions, including the cerebellum and thalamus^15^, and cortical regions such as the secondary somatosensory cortex of the parietal lobe^20^. Functional lesion studies using transcranial magnetic stimulation have found that attenuation is dependent on higher motor regions critical for preparation of action^21^, including the medial frontal cortex^22^. The presence of age-related differences in attenuation and their neural basis remain unresolved.

A robust quantitative measure of sensorimotor attenuation is the Force Matching Task^14^. In this task, participants are asked to match a target force that is applied to their index finger by a torque motor. In a control condition that examines haptic pressure perception, participants use an external device, such as a linear potentiometer (‘Slider' condition), to control the force indirectly through a torque motor. In this Slider condition, healthy adults can accurately reproduce the target force with minimal bias. However, when matching the force directly (‘Direct' condition) by pressing with the index finger of their other hand, they ‘overcompensate' with a consistently larger force^14^. This overcompensation results from sensory attenuation, and reflects the integration of predictions from internal models, based on the motor commands.

Normal ageing typically reduces sensory sensitivity, with increased sensory noise (or reduced precision) of peripheral and central processing^23^. According to the principles of Bayesian integration, reduced precision of sensory signals would lead to increased reliance on predictions. Similarly, greater precision afforded to predictive signals with ageing^24^ would also lead to increased weighting of predictions from internal models. Together, these may lead to increased sensorimotor attenuation in the Direct condition. Such altered sensorimotor attenuation with ageing may be associated with structural and functional differences in brain areas previously linked to sensorimotor integration, including medial frontal cortex and the pre-SMA^22^, the parietal cortex^20^,^25^,^26^ and cerebellum^15^,^27^.

To test these hypotheses, we used the Force Matching Task in a population-based cohort of 325 healthy adults between 18 and 88 years^28^, combined with multi-modal brain imaging of grey matter volume and functional connectivity. Our results show an age-related increase in the degree of sensorimotor attenuation, in proportion to reduced sensory sensitivity. This effect is associated with reduced grey matter volume in the pre-SMA and its functional connectivity with frontostriatal regions. These results are consistent with the hypothesis that ageing alters sensorimotor integration due to a shift in the balance between sensory signals and internal predictive models for motor control. Such a shift could contribute to the motor and cognitive changes observed with ageing.

## Results

### Demographics

Demographic details are summarized in Table 1. The number of participants was similar across the age deciles. Out of the 325 participants who performed the behavioural task, 313 participants also completed the magnetic resonance imaging (MRI) session.

### Sensory attenuation with ageing

**Figure 1:**
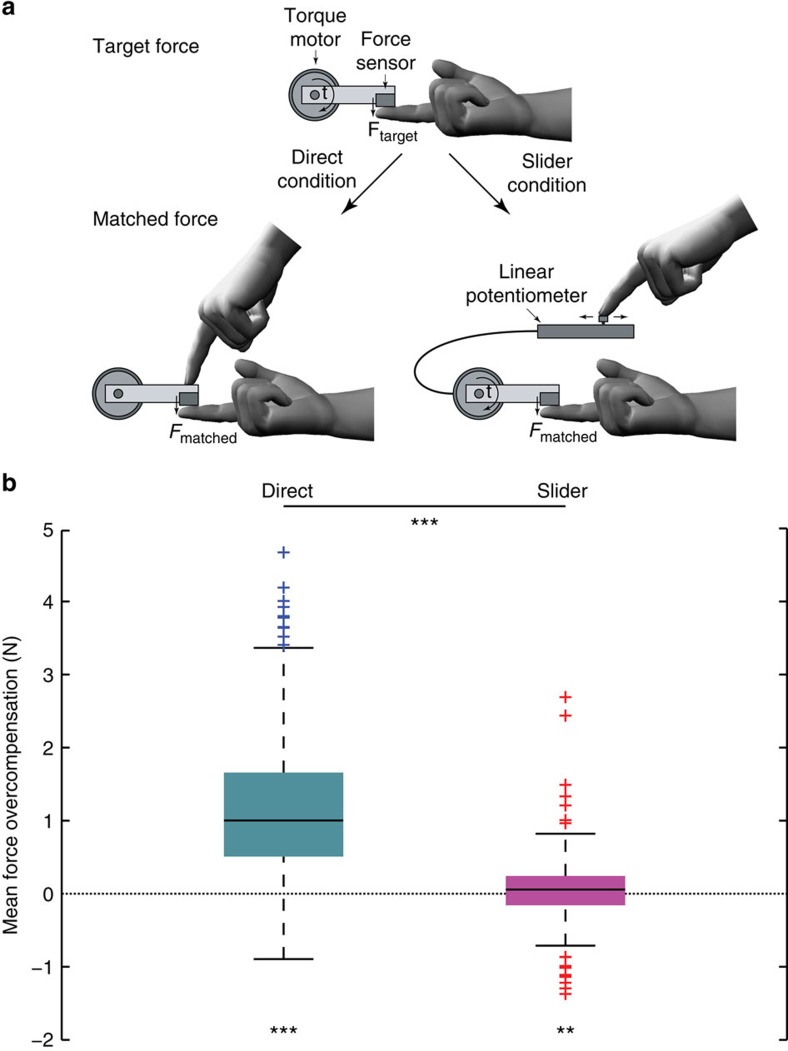
The Force Matching Task. (**a**) Schematic representation of the behavioural task, in which participants matched a force applied to their left index finger via a lever attached to a torque motor. In the Direct condition, participants used the right index finger to apply the force directly on the lever, whereas in the Slider condition, they matched the force by moving a linear potentiometer that controlled the torque motor. The main measure for each subject was the mean overcompensation, calculated as the difference between the matched force and target force across the different force levels. (**b**) Standard boxplots showing the distribution of force overcompensation values across participants in the Direct and Slider conditions (black line indicating the median). A positive value indicates sensory attenuation. Significance of comparisons is indicated by **=*P*<0.01, ***=*P*<0.001.

Across all participants, mean force overcompensation in the Direct condition was 1.198 N (s.d. 0.890 N), and was significantly greater than zero (Wilcoxon signed-rank test; *n*=322, *Z*=−15.41, *P*=1.390e−53), indicating that participants consistently attenuated the sensory consequences of their actions (Fig. 1b). Mean overcompensation in the Slider condition was far smaller than in the Direct condition (Wilcoxon two-sample paired signed-rank test; *n*=322, *Z*=−15.24, *P*=2.006e−52) with a mean of 0.052 N (s.d. 0.417 N), although still significantly greater than zero (Wilcoxon signed-rank test; *n*=322, *Z*=−2.748, *P*=0.006). Overall, participants were thus more accurate in the Slider condition than in the Direct condition in estimating and reproducing the target force. These results replicate the fundamental sensorimotor attenuation phenomenon^14^. The effect was highly robust, with 315/322 (98%) participants showing sensory attenuation of the sensory consequences of their own actions.

**Figure 2:**
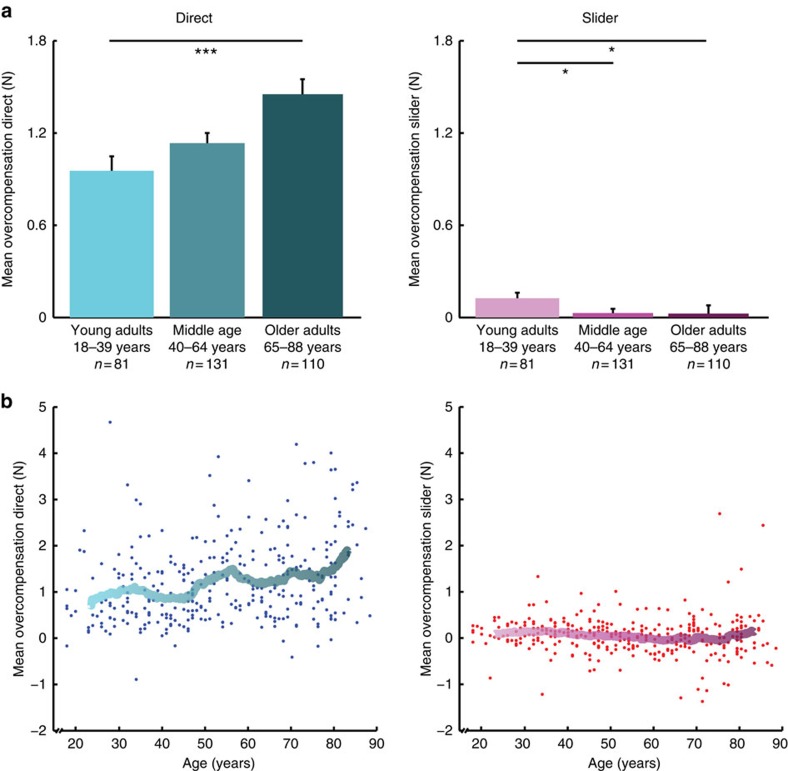
Age-related differences in sensorimotor attenuation. (**a**) Mean force overcompensation in the Direct condition (shades of cyan; left panel) and Slider condition (shades of magenta; right panel) across three age groups (error bars indicate standard error of the group mean). Significance of pair-wise comparisons is indicated by *=*P*<0.05, ***=*P*<0.001. (**b**) Overcompensation plotted against age across all participants for Direct condition (left) and Slider condition (right). For illustration, a moving average (window size of 50) is displayed.

To examine the impact of age on sensorimotor attenuation, we first split the study cohort into three groups by age, with young adult (ages 18–39), middle age (ages 40–64) and older adult (65+) groups (Fig. 2a). There was a significant effect of age on Direct force overcompensation (Kruskal–Wallis one-way analysis of variance; *n*=322, *χ^2^*=15.15, *P*=5.13e−04). *Post hoc* pair-wise comparisons showed higher Direct force overcompensation by older adults compared with young adults (Wilcoxon rank sum test; *n*=191, *Z*=3.68, *P*=6.92e-04, Bonferroni corrected). There was also a trend for higher Direct overcompensation in older adults compared with middle age (*n*=241, *Z*=2.329, *P*=0.06, Bonferroni corrected). In the Slider condition, there was also a significant effect of age (Kruskal–Wallis one-way analysis of variance; *n*=322, *χ^2^*=8.975, *P*=0.011), and pair-wise comparisons showed reduced Slider force overcompensation in older adults (*n*=191, *Z*=−2.459, *P*=0.042, Bonferroni corrected) and middle age (*n*=212, *Z*=−2.862, *P*=0.013, Bonferroni corrected) compared with young adults. Moreover, there was a significant positive correlation between age and Direct sensorimotor attenuation over the whole group (*n*=322, Spearman's rho=0.270, *P*=9.131e−07), and a weak negative correlation between age and Slider force overcompensation (*n*=322, Spearman's rho=−0.147, *P*=0.008), shown in Fig. 2b. These results suggest that sensorimotor attenuation is increased with ageing, and that this increase could not be explained by a general overcompensation bias, as older adults showed reduced overcompensation relative to young adults in the Slider condition.

**Figure 3:**
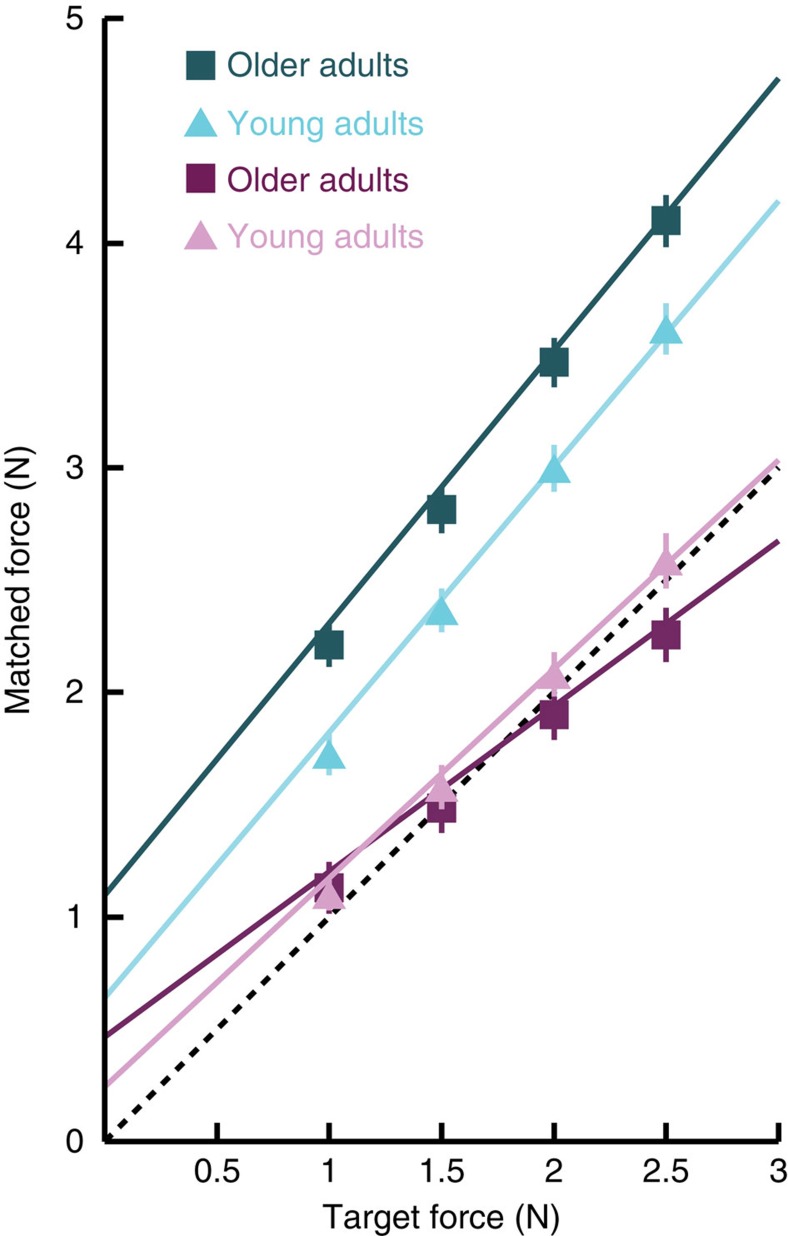
Matched force versus target force. Mean linear regression fits of the matched force against the target force for the Direct (cyan) and Slider (magenta) conditions, plotted separately for ‘young adults' (triangles) and ‘older adults' (squares). These two groups refer to the age groups described in Fig. 2a and in the main text. Mean (±2 standard error of group mean) of matched forces are shown across the different target forces for each age group. Analyses on the slope and intercept of linear regressions were performed with age as a continuous variable (see text), but are illustrated as a categorical effect for simplicity.

Changes in sensorimotor attenuation might be associated with distinct behavioural patterns in the Force Matching Task, which can be assessed by examining the relation between matched and target forces (Fig. 3). Specifically, differences in attenuation could arise from: (i) a fixed shift in overcompensation across all forces, leading to a difference in the intercept of the regression between target and matched force; or (ii) a progressive difference across forces that results in a change in the slope. To explore the origins of altered sensorimotor attenuation with age, we fit linear regression models of target force versus matched force for each participant.

Figure 3 illustrates the mean regression fits for young versus older adult groups (groups as above). The increase in Direct overcompensation was associated with an increase in the intercept with age: the intercept (*n*=322, Spearman's *ρ*=0.243, *P*=1.05e−05), but not slope (*n*=322, Spearman's *ρ*=−0.045, *P*=0.422) correlated with age. In the Slider condition, there was a reduction of the slope with ageing (*n*=322, Spearman's *ρ*=−0.278, *P*=4.155e−07), and a corresponding compensatory increase in the intercept (*n*=322, Spearman's *ρ*=0.143, *P*=0.010), so that the age-related difference in the overall overcompensation was minimal (see above).

In a set of control analyses, we confirmed that the same pattern of results persisted when considering several possible confounds, including education, gender and handedness (Supplementary Note 1); as well as the selected time window for calculating the matched forces (Supplementary Note 2 and Supplementary Table 1).

### Mechanism for differences in sensory attenuation

Increased force overcompensation in the Direct condition, explained by a positive shift in the magnitude of matched forces, indicates a consistent increase in the sensory component that was attenuated. This increase suggests an increased weighting of sensorimotor prediction signals. At the same time, there was a ‘flattening' of matched force across the different target forces in the Slider condition with ageing. This suggests that in the Slider condition, in the absence of efference copy signals used in the Direct condition, older adults were less sensitive in their basic perception of the differences between the forces (that is, they perceived the forces as more similar). This is in agreement with the reduced sensory precision observed with age^23^,^29^,^30^,^31^. We hypothesized that this reduced sensory precision contributes to an amplified weighting of sensorimotor predictions and increased attenuation in the Direct condition (see Introduction).

To test this, we first examined whether the differences in Direct intercept and Slider slope were related. There was a significant correlation between the positive shift in the intercept in the Direct condition and the flattening of slope in the Slider condition (*n*=322, Spearman's *ρ*=−0.368, *P*=1.212e−11), which was also independent of age (partial correlation; *n*=322, Spearman's *ρ*=−0.323, *P*=3.186e−09). Thus, participants who showed larger positive intercepts in the Direct condition also demonstrated a flatter slope in the Slider condition. This suggests that increased attenuation was related to reduced sensory precision.

If reduced sensory precision contributes to the age-related increase in reliance on predictions, one would expect that factoring out the Slider slope in a partial correlation would attenuate the strength of the relation between age and the Direct intercept. Indeed, factoring out the Slider slope weakened the relation between Direct intercept and age by 35% (proportion of Spearman's *ρ* reduced when including Slider slope in a partial correlation). Moreover, the shared variance of Slider slope explained 64% of the total variance of the linear relation between Direct intercept and age (calculated as 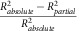). A similar pattern of results was found when the variability of matched forces in the Slider condition was used as a measure of sensory precision (Supplementary Note 3). These results suggest that reduced precision of sensory signals partly contributes to the increased weighting of sensorimotor predictions with ageing.

### Brain substrates for differences in attenuation with ageing

**Figure 4:**
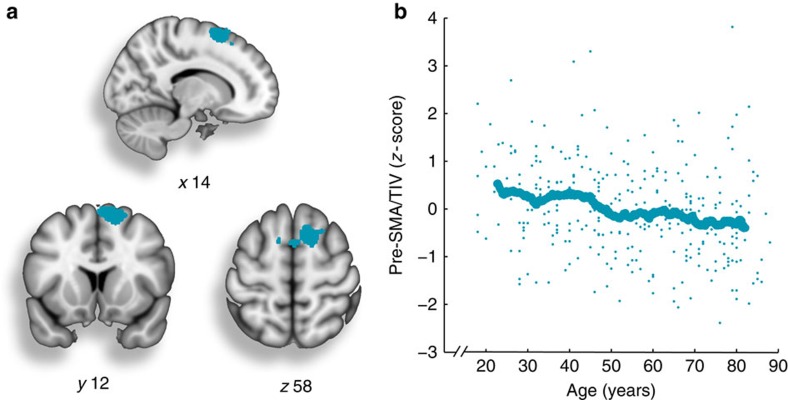
Differences in grey matter volume in relation to sensorimotor attenuation. (**a**) Results of a Voxel-Based Morphometry analysis, examining the correlation between grey matter volume and Direct force overcompensation. Grey matter volume in the pre-SMA (peak voxel in *x*=+14, *y*=+12, *z*=+58) was negatively associated with increased sensorimotor attenuation in the Direct condition (cyan; *P*<0.05, FWE-corrected with cluster-forming threshold of *P*<0.001, uncorrected). Cluster size=1,193 voxels; voxel size=3.375 mm^3^. (**b**) The *z*-score of grey matter in the pre-SMA cluster from **a** divided by total intracranial volume, plotted as a function of age. The pre-SMA cluster also demonstrated an age-related difference in grey matter volume with (*n*=302, *r*=−0.25, *P*=1.2e−05) and without (*n*=302, *r*=−0.29, *P*=2.1e−07) adjusting for total intracranial volume. For illustration, a moving average (window size of 50) is displayed.

To examine the brain network differences associated with increased sensorimotor attenuation with ageing, we first identified the structural correlates of sensorimotor attenuation. This allowed us to identify a specific region for a seed-based functional connectivity analysis without making *a priori* regional assumptions. A whole-brain Voxel-Based Morphometry (VBM) implementing a multiple regression analysis revealed one cluster in the pre-SMA (peak voxel *x*=+14, *y*=+12, *z*=+58; *n*=302, *P*<0.05, Family Wise Error (FWE) corrected; Fig. 4a), which was negatively associated with Direct force overcompensation. Although this cluster also independently showed an age-related reduction in grey matter (Fig. 4b), this regression analysis showed that Direct force overcompensation and pre-SMA were negatively correlated over and above the effect of age. That is, across all participants, increased sensorimotor attenuation was related to lower grey matter volume in the pre-SMA cluster. No areas showed a positive correlation with Direct force overcompensation, nor any correlation with force overcompensation in the Slider condition. For completeness, a subsidiary analysis showed no areas associated with the interaction between age and Direct force overcompensation.

The pre-SMA cluster showing an association with attenuation in the VBM above was used as a seed for functional connectivity analyses on continuous time series of BOLD signal. Functional MRI (fMRI) was acquired during a movement task^32^,^33^ and a task-free ‘resting state'. The cued movement task activated widespread frontoparietal regions of the sensory and motor systems (Supplementary Methods and Supplementary Fig. 1A), and we confirmed that these regions included the pre-SMA seed to be used for the functional connectivity analysis (Supplementary Fig. 1B). Further, the resting state scans allowed us to examine connectivity that is not confined to movement or to a specific task setup.

We used a conjunction analysis to identify clusters whose connectivity with the pre-SMA seed showed effects of both ageing and increased Direct force overcompensation. During the movement task, reduced connectivity as a function of ageing and Direct overcompensation was found with several regions, including supramarginal gyrus of the posterior parietal cortex, bilateral prefrontal and striatum (Supplementary Table 2; *n*=280, *P*<0.05, FWE-corrected). During resting state, reduced connectivity was also found in the bilateral prefrontal and striatal regions, as well as in the cerebellum and medial temporal lobe (Supplementary Table 3; *n*=280, *P*<0.05, FWE-corrected). Significant increases in connectivity were found in the movement task and resting state in the postcentral gyrus. There were no areas where pre-SMA functional connectivity correlated with Slider overcompensation, and a subsidiary analysis showed no significant clusters showing an interaction between age and Direct force overcompensation.

**Figure 5:**
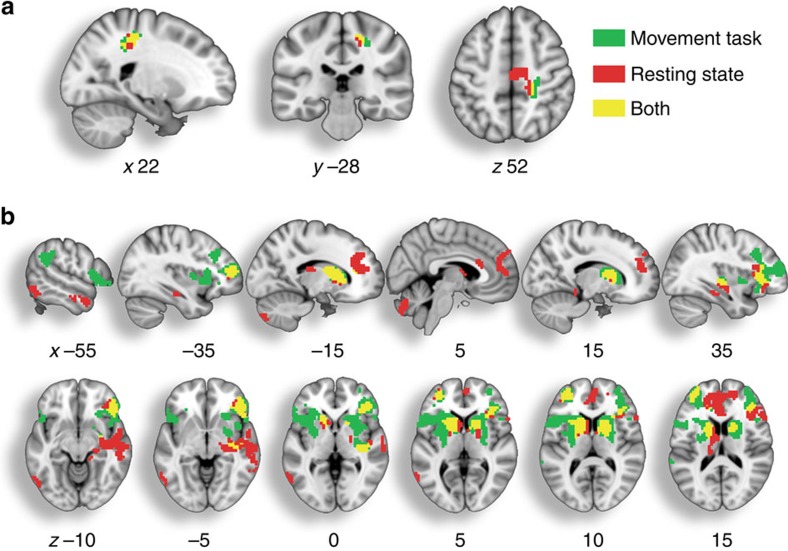
Differences in functional connectivity with pre-SMA in relation to ageing and increased Direct force overcompensation. (**a**) Clusters showing increased functional connectivity with the pre-SMA seed from the VBM analysis in relation to ageing and Direct force overcompensation, during a movement task (green), during task-free resting state (red) or both (yellow). Significant (*P*<0.05, FWE-corrected) clusters in each of the two fMRI datasets were identified at a cluster forming threshold of *P*<0.001, uncorrected. (**b**) Same as in **a**, but for clusters showing reduced connectivity with pre-SMA in relation to ageing and increased Direct overcompensation.

Figure 5 shows the clusters exhibiting a significant conjunction of age and Direct overcompensation from the movement task, from the resting state and their overlap, which is summarized in Table 2. There was one overlapping cluster in the primary somatosensory cortex that showed increased connectivity with the pre-SMA in relation to both ageing and increased attenuation (Fig. 5a). Five overlapping clusters showed reduced connectivity with the pre-SMA in relation to ageing and attenuation, including bilateral prefrontal cortex, caudate, putamen and insular cortex (Fig. 5b). Consistent with these findings, all five overlapping clusters from the two fMRI data sets also showed reduced grey matter volume in relation to increased sensorimotor attenuation and ageing (Supplementary Fig. 2). Taken together, these results suggest that older participants show exaggerated sensorimotor attenuation in association with reduced grey matter volume and reduced connectivity of the pre-SMA in frontostriatal circuits.

## Discussion

The principal result of this study is that older adults showed increased sensorimotor attenuation. Structural and functional imaging data indicate that the effect of age on sensorimotor attenuation is related to differences in the pre-SMA in terms of its volume and its connectivity with prefrontal and striatal regions. We propose that reduced structural integrity of the pre-SMA and its connectivity within frontostriatal circuits, lead to a shift in the balance between internal prediction signals and the sensorium.

To study age-related differences in internal predictive models and their integration with sensory feedback, we used the Force Matching Task^14^. The task provided a robust measure of sensorimotor attenuation, which we observed as force overcompensation in 98% of participants in the Direct condition. The attenuation of sensations arising from one's own action depends on the integration of predictive signals with sensory feedback^13^. Increased attenuation with ageing therefore suggests a stronger weighting of predictive signals for sensorimotor integration. Interestingly, although the overall estimated bias was minimal in the Slider condition (0.05 N), older adults were more accurate than young adults when matching the forces in the Slider condition. The combination of a positive association between ageing and overcompensation in the Direct condition with a negative association in the Slider condition is not readily explained by a general compensatory process or cognitive strategy (Supplementary Note 4).

We examined the mechanism for these distinct behavioural differences with age by looking at the relation between matched and target forces. In the Direct condition, there was a consistent increase in attenuation across the different target forces, reflected by an increased intercept of the linear regression with age. By contrast, in the Slider condition there was a reduced slope of the linear regression with age, suggesting reduced basic perceptual sensitivity to the differences between forces, which is likely to reflect the reduced sensory precision observed with age^23^. The reduction in Slider slope was not only correlated with the degree of sensorimotor attenuation (over and above the effect of age) but also explained a large proportion of the relationship between attenuation and age. A similar pattern was found when using Slider overcompensation variability as a measure that reflects sensory precision. These selective effects on the slope and intercept are again inconsistent with a general compensatory response to altered sensory thresholds (see Supplementary Note 4). Instead, they suggest that reduced sensory precision with age contributes in part to the over-weighting of prediction signals for movement and increased attenuation.

The increase in Direct intercept and reduction in Slider slope both support the hypothesis that ageing is associated with an increased weighting of predictive models, while down-weighting sensory information. Generally, the combination of sensory and predictive signals adheres to optimal precision-weighted Bayesian integration^8^, such that their weight depends on their relative precision (that is, the inverse of their variance, or noise). In the Slider condition, the perception of force would depend on a combination of sensory evidence and prior. As the sensory evidence becomes more variable (that is, less precise or with increased sensory noise) with age, participants rely more on a prior, which in this case is likely related to the average force experienced over the session. The reduced sensitivity and ‘flattening' of the target-matched force relation around the mean target force (see Fig. 3) suggest less influence of sensory input on force perception in each trial as a result of increased weighting on the prior (average target force). This may have made older adults overall more accurate in the Slider condition compared with young adults.

In contrast to the Slider condition, in the Direct condition participants can use an efference copy of their motor command to predict the perceived force they apply themselves^15^. As sensory variability increases and experience accumulates with age, older participants may rely more on internal predictive models. This would result in greater sensory attenuation, leading to increased overcompensation across the entire range of forces in the Direct condition. The age-related increase in reliance on the efference copy may dominate the effect of age on the prior (average force) that was observed in the Slider condition. Both observations could therefore arise from a noisier sensory system, which is degraded with age^29^,^30^,^31^, and an increased precision of sensorimotor prediction owing to the accumulation of experience throughout life^24^. We propose that these age-related changes lead to a shift in the balance between the sensorium and internal models in sensorimotor integration.

The greater relative increase in the precision of prediction signals with age could be an adaptive mechanism for healthy ageing^24^. An advantage for enhanced attenuation with age would be a preservation of the sense of agency (the sense that one controls one's own actions and their consequences) and the relative salience of external stimuli^34^. Indeed, reduced sensorimotor attenuation has been linked to impaired awareness of action and mental health disorders such as schizophrenia^16^ and psychogenic movement disorders^17^. Unless offset by greater weighting of internal models based on experience, noisy sensory information with ageing would otherwise lead to a diminished ability to distinguish between self- and externally caused sensations, and consequently an abnormal attribution of action.

Another advantage for greater weighting of internal predictions in motor control is the minimization of unnecessary error-based motor learning. Motor learning is achieved through the updating of internal models in the context of movement errors—that is, the discrepancy between one's goal and one's current state^8^. In an uncertain environment, where sensory noise is greater, an imprecise sensory system, such as that seen in old age, would lead to an unreliable estimation of one's current state and the incorrect detection of movement errors that would call for excessive movement adaptation. Down-weighting the noisy sensorium would minimize or slow the adjustment of movements and promote consistency in performance^35^.

The reduced impact of sensory input on sensorimotor integration may also have disadvantages. For example, it may reduce the updating of internal models^36^, leading to impaired motor learning with age^4^. Motor learning^37^ and cognitive tasks^38^ are thought to combine model-based and model-free mechanisms. The reduced adaptation of internal models with age may promote a compensatory shift towards model-free strategies and a greater reliance on feedback-driven corrections during movement^39^. This concept of a greater dependence on external cues and feedback-based strategies has been previously suggested to contribute to many cognitive changes with age^40^. In the motor domain, reduced sensitivity to the sensorium and altered sensorimotor integration as suggested by the current study may also contribute to the decline in motor function in old age. This has important social and economic implications, as the prevalence of disorders of motor control and their consequences (for example, falls) are set to rise rapidly with the ageing of the population^41^,^42^,^43^, which poses an increasing health burden^6^.

In addition to the behavioural results, we found that grey matter volume in the pre-SMA correlated negatively with sensorimotor attenuation. In previous studies, additional brain regions have been linked to sensory attenuation, including frontal^22^ and parietal cortex^20^,^25^,^26^, as well as the cerebellum and thalamus^15^,^27^. We suggest that sensorimotor attenuation arises from a complex interaction between cortical and subcortical brain areas; an interaction that we examined using seed-based fMRI analyses.

We examined the functional connectivity of the pre-SMA with fMRI. We used a simple movement task and resting state scan as the force matching task is not easily adapted to the MRI environment. The movement task was aimed to evoke brain activity in key sensorimotor areas^33^, allowing us to examine the correlation between fluctuations in regional fMRI signals relevant to movement, in relation to ageing and sensorimotor attenuation. We adopted a similar approach for analysing connectivity in relation to ageing and attenuation during task-free resting state. These separate analyses revealed similar increases in pre-SMA connectivity with primary somatosensory cortex in relation to increased attenuation with ageing. This suggests that the pre-SMA modulates the perceived intensity of one's action^22^ by adjusting activity in the somatosensory cortex. The analyses of fMRI both at rest and during movement confirmed changes in the connectivity of the pre-SMA in association with both increased attenuation and ageing. Specifically, there was reduced connectivity of the pre-SMA with the lateral prefrontal and frontopolar cortex, caudate, putamen and insula. These regions also showed reduced grey matter volume in the older participants who showed increased attenuation.

These frontostriatal circuits are central to normal motor control^44^,^45^,^46^. Input from the striatum to the medical frontal cortex provides signals for the initiation of a volitional action^47^, together with cerebellar input^48^. The cerebellum is believed to generate prediction signals, such as an efference copy, to the medial frontal cortex, which are relayed to the striatum before movement initiation^45^. The insula may integrate these sensorimotor signals for high-level awareness of action^49^. Lastly, the lateral prefrontal cortex, including the frontopolar cortex, could select an action based on internal goals, by providing hierarchically ordered control signals^50^. Reduced coherence in this network is associated with impairments in voluntary action^51^.

The caudate and putamen, which showed reduced connectivity with pre-SMA in association with enhanced sensorimotor attenuation with ageing, become significantly depleted of dopamine even in normal ageing^52^. Such reduced dopaminergic modulation could facilitate a shift towards model-free performance^53^ as may occur in old age^40^ (see above). Moreover, change in dopaminergic modulation of cortico-striatal circuits is proposed to result in a ‘noisier' representation of information^54^, and altered precision of prediction signals^12^. Thus, age-related reduction in striatal dopamine could lead to a diminished capacity of frontostriatal circuits to flexibly modulate the precision of prediction signals, leading to a consistently increased weight afforded to prior predictions. Altered dopaminergic transmission may thereby provide a neuromodulatory mechanism, accounting for a range of motor and cognitive changes observed in healthy ageing^55^.

Along with differences in frontostriatal connectivity, there was reduced functional connectivity between the pre-SMA and posterior parietal cortex during the movement task. The pre-SMA and posterior parietal regions are hubs for internal and external action systems^47^, respectively^51^,^56^: while these regions are strongly interconnected, the former is also closely connected with the striatum, prefrontal and medial frontal cortex in association with internally triggered movement, whereas the latter is more closely associated with movements driven by environmental stimuli through a parietal-lateral premotor network^47^. The reduced connectivity we observed between pre-SMA and parietal cortex suggests a functional disconnection, affecting the interaction between these action systems during movement. The correlation method we used, however, is not able to determine the direction of influence between these regions.

Several potentially confounding factors must also be considered, including (i) memory differences, (ii) movement speed and kinematics and (iii) sensory deficits. First, short-term memory deficits are common in the ageing population^57^, and might have affected the ability of older participants to store and retrieve the target force. Compared with other psychophysiological methods, the Force Matching Task is less demanding on short-term memory, and can exclude consistent memory biases, since in a force matching paradigm any systematic bias is factored out. Importantly, memory requirements were the same in the Direct and Slider conditions. As older participants were more accurate in the Slider condition, it is unlikely that age-related differences in Direct overcompensation could be accounted for by memory deficits.

Second, although maximum response time was limited in the experiment, movement speed and kinematics could affect the applied force over time, especially in ageing^58^. We therefore re-examined the force traces and further calculated matched forces in different time windows. These additional analyses confirmed no significant relationship between age and the difference between matched forces across time windows. Critically, the same pattern of results was obtained with different time windows. These analyses suggest that, although movement speed and kinematics are likely to vary across age, they have not significantly contributed to the main results.

Third, sensory deficits are common in ageing^23^ and one must consider the effect that this might have on task performance. By using the Force Matching Task, we minimize the impact of such changes, as consistent biases are factored out. For example, increased peripheral sensory thresholds or reduced intensity of afferent signals would not normally bias the measured force overcompensation (Supplementary Note 5). Sensory deficits with ageing were observed in our cohort, as reduced sensitivity to force differences in the Slider condition (the slope). These differences could partly explain the relation of sensory attenuation in the Direct condition and age. Importantly, reduced slope in the Slider condition was related to a reduced Slider overcompensation. Thus, age-related sensory differences were manifest as opposite effects on Direct and Slider overcompensation, suggesting that sensory deficit alone does not lead to a general increase in force overcompensation.

In summary, this large population-based study of healthy ageing found that older participants show enhanced sensorimotor attenuation, which can be explained as a shift in motor control from noisier sensory information towards internal prediction signals. This phenomenon was associated with reduced grey matter volume in the pre-SMA and its reduced functional connectivity with a frontostriatal network. Although our study was confined to motor control, this mechanism may have broader implications for understanding the effects of healthy ageing on other cognitive domains, such as perception and learning.

## Methods

### Participants and force matching task

A population-based cohort of healthy adults (*n*=325) was recruited as part of the Cambridge Centre for Ageing and Neuroscience (Cam-CAN)^28^,^33^. The study was approved by the Cambridgeshire 2 (now East of England—Cambridge Central) Research Ethics Committee, and all participants provided a written informed consent prior to the study. Participants performed the Force Matching Task (see Fig. 1a)^14^. This task implements the psychophysical technique known as the Method of Adjustment, in which participants adjust the level of the stimulus to match a previously presented stimulus. On each trial, a lever attached to a torque motor applied the target force to the left index finger, which rested under the lever. The target force was chosen from the set: 1.0, 1.5, 2.0 and 2.5 Newtons (N). Within a cycle of four trials, each target force was presented once in a pseudo-random order. The target force was presented for 2.5 s, and was ramped up (and down) linearly over 0.25 s before (and after) the presentation period. At the end of the presentation period, the force was removed (lever released) and participants used their right index finger to match the force they had just perceived on their left finger. Unless stated otherwise, the matched force was calculated as the mean force measured between 2 and 2.5 s after the start of the matching period, as in previous studies^14^,^17^.

There were two conditions. In the Direct condition, participants matched the target force by pressing directly on top of the lever, mechanically transmitting the force to the left finger. In the Slider condition, participants matched the force with their right index finger by moving a slider (a linear potentiometer) which controlled the torque motor. A force sensor at the end of the lever measured both the target and matched forces applied to the left finger. The Slider condition allows the evaluation of sensory biases in the force matching procedure, as well as the estimation of variability in basic tactile perception. All participants performed both the Direct and Slider conditions, the order of which was counterbalanced across participants. For each condition, an initial familiarization phase of eight trials (two cycles of the four target forces) was performed. The main experiment for each condition consisted of 32 trials (eight cycles).

Mean overcompensation was calculated as the average difference between the matched and the target force on each trial across the four target force levels. Three participants were excluded from behavioural and neuroimaging analyses, as their mean Direct overcompensation value was larger than 3 s.d. from the entire study group mean, leaving 322 participants for the behavioural data analyses. In addition to mean overcompensation, the intercept and slope of the matched versus target force linear regression were calculated for each subject in order to further examine differences in task performance. Statistical analyses of the behavioural data were performed using non-parametric tests, unless stated otherwise, and implemented with R (ref. 59).

### Structural imaging

We scanned 313 participants (12 declined MRI) with a 3T Siemens TIM Trio System, employing a 32 channel head coil. T1-weighted MPRAGE images were acquired (TR 2,250 ms, TE 2.99 ms, TI 900 ms, FA 9°, FOV 256 mm × 240 mm × 192 mm, isotropic 1 mm voxels). Both the structural and functional (see below) images were preprocessed with the automatic analysis batching system (http://imaging.mrc-cbu.cam.ac.uk/imaging/AA) using SPM12 (ref. 33). The structural images were preprocessed for a VBM analysis. SPM12 segmentation was used for less age-biased tissue priors. Diffeomorphic Anatomical Registration Through Exponentiated Lie Algebra (DARTEL) was applied to improve inter-subject alignment^60^ as follows: segmented images from all the participants who were scanned in the Cam-CAN project (*n*=651) were normalized into a project-specific template. The resulting images relevant for this study were then normalized to the Montreal Neurological Institute (MNI) space, followed by modulation and smoothing with an 8-mm full-width at half-maximum Gaussian kernel. The MRI data of eight participants were not included in the analysis due to technical problems during the scanning session, preprocessing or brain structural abnormalities. After exclusion of three participants due to their behavioural data (see above), 302 participants had valid structural neuroimaging data.

The aim of the structural imaging analysis was to adopt an unbiased whole-brain method to identify a brain region specifically related to sensorimotor attenuation (over and above age), so as to use it as a seed for the functional connectivity analyses (below). Multiple regression analysis was performed to create a statistical parametric map of differences in grey matter volume in relation to sensorimotor attenuation. For the analyses, an inclusive mask of an absolute threshold of 0.15 was used on the preprocessed images for the inclusion of grey matter voxels. Direct force overcompensation was included as the covariate of interest. Covariates of no interest included Slider overcompensation, age (as a continuous variable), scanner coil (before versus after a change in gradient coil required in the scanner), handedness (entered as the Edinburgh handedness score^61^) and gender. In a subsidiary analysis, the orthogonalized interaction term of Direct overcompensation and age was included as an additional covariate. All variables were mean-corrected and z-score scaled before entry into the regression analyses. Significant clusters were identified at *P*<0.05, FWE corrected, with a cluster-forming threshold of *P*<0.001, uncorrected.

### Functional imaging

At the same scanning session, the same participants were also scanned for T2-weighted functional MRI (fMRI) data using a Gradient-Echo Echo-Planar Imaging sequence (TR 1,970 ms, TE 30 ms, FA 78°, FOV 192 mm × 192 mm, 3 mm × 3 mm × 4.44 mm voxel size). In total, 261 volumes were acquired (first five discarded for T1-equilibration), each containing 32 axial slices (descending order) of 3.7 mm thickness (20% interslice gap).

These functional images were acquired during a simple movement task and task-free ‘resting state'. In the movement task, participants pressed a button in response to audio-visual cues^33^. Participants responded to the display of two circular checkerboards presented for 34 ms to the left and right of a central fixation cross and a simultaneous presentation of a binaural tone (300 ms duration; 300, 600 or 1,200 Hz frequencies pseudorandomly ordered with equal numbers of trials for each frequency). In addition to 120 (plus one practice) audio-visual trials, there were four ‘visual only' trials and four ‘auditory only' trials. These unimodal trials were included so as to minimize the strategic responses to a specific modality while ignoring the other modality. Participants were asked to press a button with their right index finger whenever they heard and/or saw the stimuli in the 128 stimulus trials. Speed was not emphasized. These stimulus trials were combined with 127 null trials of the same length during which a fixation cross was displayed in the centre of the screen. The order and timing of all trials were pseudorandomly determined so as to optimize functional MRI signal, using a 255-length ‘m-sequence'^62^ with *m*=2 and minimal stimulus onset asynchrony of 2 s (stimulus onset asynchrony ranging from 2 to 26 s). The task was 8 min and 40 s long. At the same scanning session, using the same scanning protocol, task-free resting state scans were acquired, during which participants rested with their eyes closed for 8 min and 40 s; the same duration as the movement task.

Preprocessing and analysis steps were the same across scan types (movement task and resting state) unless otherwise stated. The preprocessing and minimization of motion effects followed Geerligs *et al*.^63^. The functional T2*-weighted echo planar images were corrected for distortions using fieldmaps, and were motion- and slice-time corrected with SPM12. The mean echo planar images were then co-registered with the T1 image, and normalized to MNI space using the same normalization parameters determined via the DARTEL template above. A wavelet despiking method was then applied to reduce motion artefacts^64^. Participants with a mean spike percentage greater than 2 s.d. of the group mean were excluded from the analysis; based on this criterion, 22 out of the 302 participants with both valid structural and functional datasets were excluded (7.3%). The remaining 280 ‘despiked' functional images were included in the analyses after smoothing with an 8 mm full-width at half maximum Gaussian kernel.

To identify age-related differences in a network associated with sensorimotor attenuation, a seed-based functional connectivity analysis was performed, using the results of the main VBM analysis above as a seed region. Specifically, the seed region was a 10 mm sphere centred on the peak voxel where grey matter volume correlated with Direct overcompensation. The first-level analysis picked out voxels whose time series positively correlated with the mean time series of the seed (that is, time series averaged across voxels within the seed) for each participant. The analysis included additional parameters for reducing motion artefacts: (1) the six original motion parameters; (2) mean signal from white matter tissue segment (after despiking and before smoothing), where the segment included voxels with less than 1% grey matter and more than 80% white matter; (3) same as 2 but for cerebrospinal fluid segment; (4) first-order temporal derivative, squares and squared derivatives of 1–3 (ref. 65); (5) a discrete cosine basis set, implementing a band-pass filter from 0.009 to 0.1 Hz. The resulting beta images for each participant were entered into a second-level analysis, which included Direct force overcompensation and age as the covariates of interest; as well as the following covariates of no interest: Slider overcompensation, handedness (Edinburgh handedness score^61^), scanner coil (before versus after coil change), gender and total motion, computed as the root mean square volume-to-volume displacement^66^. In the movement task analysis, participant's mean reaction time was included as an additional covariate. In a subsidiary analysis, the orthogonalized interaction term of Direct overcompensation and age was included as an additional covariate. All variables were mean-corrected and *z*-score scaled before entered into the regression analyses.

To identify clusters that showed changes in functional connectivity with both ageing and increased Direct force overcompensation, a conjunction analysis was performed on the combined effects of Direct overcompensation and age, tested against global null hypothesis. Clusters were identified at *P*<0.05, FWE-corrected, with a cluster-forming threshold of *P*<0.001, uncorrected. Commonalities *across* the two fMRI data sets (movement task and resting state) were found by identifying voxels that (1) survived *P*<0.001, uncorrected, in both datasets; and (2) were part of clusters whose spatial extent survived *P*<0.05, FWE-corrected in the above conjunction of age and Direct overcompensation ‘within' either fMRI dataset. Significant clusters were labelled according to the Harvard-Oxford and Juelich probabilistic atlases in FSL (http://fsl.fmrib.ox.ac.uk/fsl/).

### Data availability

The raw data and analysis code are available upon signing a data sharing request form (http://www.mrc-cbu.cam.ac.uk/datasets/camcan/).

## Additional information

**How to cite this article:** Wolpe, N. *et al*. Ageing increases reliance on sensorimotor prediction through structural and functional differences in frontostriatal circuits. *Nat. Commun.* **7**, 13034 doi: 10.1038/ncomms13034 (2016).

## Supplementary Material

Supplementary Information Supplementary Figures 1 – 2, Supplementary Tables 1 – 3, Supplementary Notes 1 – 5 and Supplementary Methods

## Figures and Tables

**Table 1:** Summary of participant demographics across age deciles.

Age	*N*	Gender M/F	Handedness R/L	Education*			
				None	GCSE	A Levels	University
18–29	34	21/13	31/3	0	5	6	23
30–39	47	24/23	41/6	1	3	5	38
40–49	59	28/31	50/9	4	9	0	46
50–59	47	25/22	42/5	3	10	10	24
60–69	56	31/25	51/5	4	15	7	30
70–79	53	21/32	49/4	6	10	5	32
80–88	29	16/13	28/1	5	7	6	11
Total	325	166/159	292/33	23	59	39	204

^*^Categorized according to the British education system: ‘none', no education over the age of 16 years; ‘GCSE', General Certificate of Secondary Education; ‘A Levels', General Certificate of Education Advanced Level; ‘University', undergraduate or graduate degree.

**Table 2:** Clusters showing differences in functional connectivity with pre-SMA in relation to ageing and increased Direct overcompensation during both movement and resting state.

Cluster region		Size
*Increased connectivity*		
R	Primary somatosensory cortex	20
	
*Reduced connectivity*		
R	Frontal pole, middle frontal gyrus and inferior frontal gyrus	207
L	Caudate and putamen	157
R	Caudate and putamen	92
L	Frontal pole	81
R	Posterior insula and putamen	53

Abbreviations: fMRI, functional magnetic field resonance; FWE, Family Wise Error; L, left hemisphere; R, right hemisphere.

Significant overlapping clusters (*n*=280, *P*<0.05, FWE-corrected) found at cluster forming threshold of *P*<0.001, uncorrected, in each fMRI data set (movement task and resting state), ordered by cluster size (in number of voxels), where each voxel=27 mm^3^.
