# Supplementary material for: Ageing increases reliance on sensorimotor prediction through structural and functional differences in frontostriatal circuits
Source: Nat Commun. 2016 Oct 3;7:13034. doi: 10.1038/ncomms13034 (PMC5063954; doi:10.1038/ncomms13034)
Supplement: Supplementary Information — Supplementary Figures 1 – 2, Supplementary Tables 1 – 3, Supplementary Notes 1 – 5 and Supplementary Methods [file ncomms13034-s1.pdf]

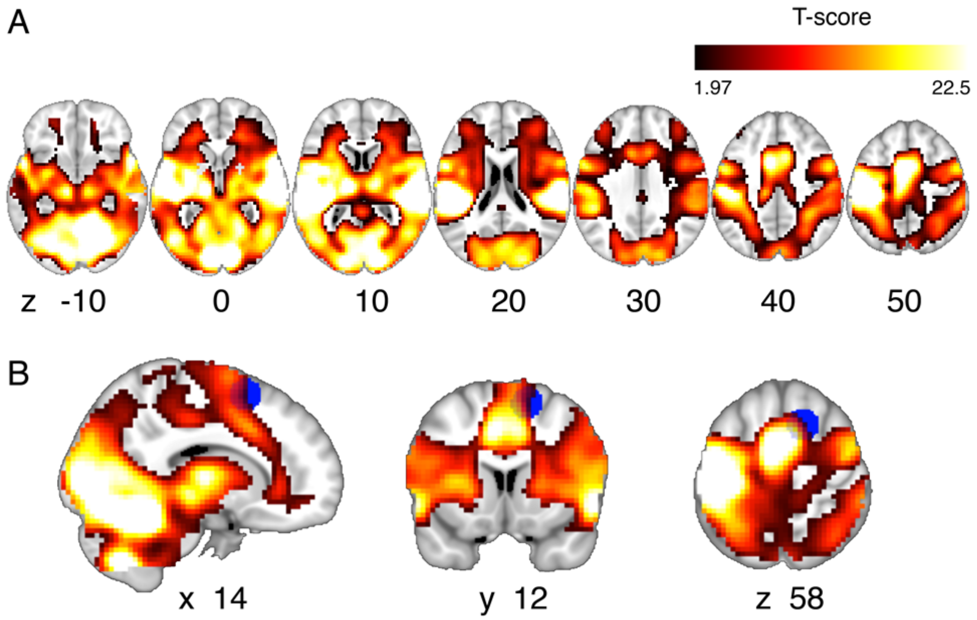

SUPPLEMENTARY FIGURE 1 | BOLD activity during the movement task and its relation to the seed region for the functional connectivity analyses. (A) Whole-group main effect of stimuli against null trials, thresholded at T=1.97 (which was equivalent to Z=1.96 with n=278). The task activated a large network of sensorimotor regions, including frontoparietal areas and the cerebellum. (B) Overlap between brain regions showing increased BOLD signal during the fMRI movement task and pre-SMA seed region (blue) derived from the VBM analysis (see main text). Slices shown are for peak voxel coordinates of the VBM analysis. Overall, 62.4% of the seed volume overlapped with the thresholded t-map shown in (A) with a mean t-value of 9.81 within the overlapping voxels. This overlap motivated us to look at functional connectivity of the pre-SMA seed region during the movement task, and to examine how this connectivity varied in relation to differences in sensory attenuation and ageing.

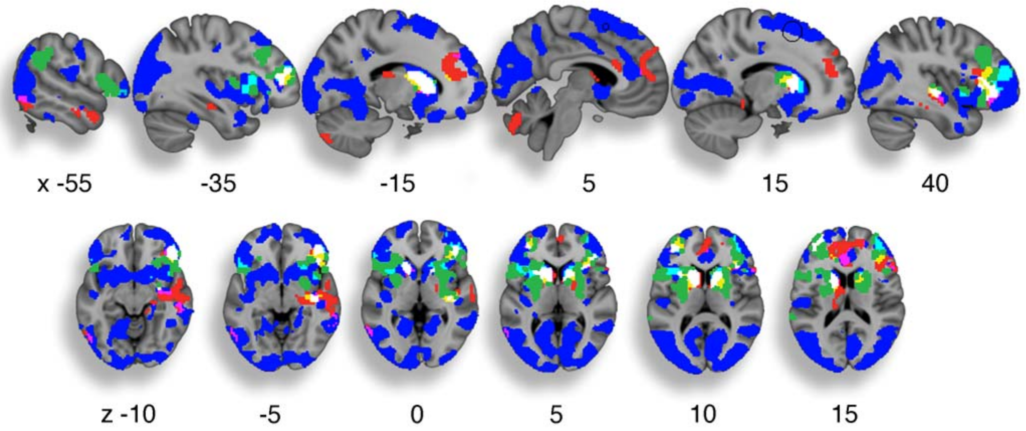

SUPPLEMENTARY FIGURE 2 | Reduction in grey matter volume and functional connectivity in relation to sensorimotor attenuation and age. Conjunction analysis of the combined effects of increased attenuation and age on grey matter volume. Significant clusters (displayed in blue) were identified at $p<0.05$, FWE-corrected ($n=280$), with a cluster-forming threshold of $p<0.001$, uncorrected. The significant clusters showed a 79% overlap with the pre-SMA functional connectivity seed (demarcated by a black line), with a mean t-value of 3.16 within the overlapping voxels. These are overlaid with clusters found in the functional connectivity analyses during movement (green) and resting state (red). The overlaps between the clusters are illustrated as a combination of red, green and blue. All five clusters that were consistent for both fMRI datasets (movement task and resting state) also showed reduced grey matter volume in relation to attenuation and ageing (overlap between the three datasets displayed in white).

SUPPLEMENTARY TABLE 1 | Control analyses of different time windows for calculating the matched forces.

Correlation / Time window		Age and force overcompensation (Spearman's rho, *p-value*)	Age and target-matched force intercept (Spearman's rho, *p-value*)	Age and target-matched force slope (Spearman's rho, *p-value*)
2.5-3 s	Direct	***0.27, 8.15e-07	***0.24, 2.03e-05	-0.04, 0.48
3-3.5 s		***0.27, 7.84e-07	***0.24, 1.46e-05	-0.03, 0.55
2.5-3 s	Slider	*-0.14, 0.01	*0.14, 0.01	***-0.27, 1.08e-06
3-3.5 s		*-0.14, 0.01	*0.13, 0.02	***-0.26, 3.16e-06

Coefficients (Spearman's rho) and *p-values* for the correlations between age and 1) force overcompensation; 2) intercept of matched versus target force regression; and 3) slope of matched versus target force regression. These are shown for two additional time windows for both Direct and Slider conditions. The significant correlations in the main text are consistent across the different time windows that were analysed. Significance levels indicated by ***=p<0.001; *=p<0.05 (n=322).

SUPPLEMENTARY TABLE 2 | Clusters showing differences in functional connectivity with pre-SMA in relation to ageing and increased Direct overcompensation during the movement task

**Brain region**		K_E	**Coordinates**			**T-score**
			**x**	**y**	**z**	
**Reduced connectivity**						
R	Caudate	1121	15	15	9	3.66
	Frontal orbital cortex		45	33	-3	3.49
	Caudate		18	3	15	3.44
L	Caudate	748	-18	15	9	3.29
	Frontal operculum cortex		-39	15	0	3.29
	Inferior frontal gyrus		-57	15	6	3.14
L	Frontal pole	308	-36	51	15	3.89
	Middle frontal gyrus		-33	24	30	3.22
	Frontal pole		-24	36	18	2.57
L	Supramarginal gyrus	123	-54	-48	30	4.06
	Superior temporal gyrus		-66	-39	12	2.17
**Increased connectivity**						
R	Primary somatosensory cortex	94	18	-30	48	2.61
	Precentral gyrus		6	-18	48	2.58
	Superior parietal lobule		21	-39	42	2.13

Significant (n=280, p<0.05, FWE-corrected) clusters, identified at p<0.001, uncorrected, ordered by cluster size K_E (each voxel=27 mm^3). For completeness, we also report the clusters' peak voxels and their T-scores. L=left hemisphere; R=right hemisphere

SUPPLEMENTARY TABLE 3 | Clusters showing differences in functional connectivity with pre-SMA in relation to ageing and increased Direct overcompensation during resting state

Brain region		K_E	Coordinates			T-score
			X	y	z	
**Reduced connectivity**						
L	Frontal pole	643	-33	54	12	3.49
	Paracingulate gyrus		-18	39	15	2.94
	Cingulate gyrus		-3	21	21	2.93
R	Middle temporal gyrus	610	60	-24	-9	3.5
	Middle temporal gyrus		54	-15	-18	3.27
	Caudate		15	9	9	3.11
R	Frontal pole	326	42	36	-3	3.58
	Inferior frontal gyrus		39	24	15	2.93
	Insular cortex		33	24	6	2.51
L	Caudate	245	-12	9	9	3.51
	Thalamus		-12	-27	15	2.05
R	Cerebellum	96	3	-75	-33	2.99
L	Cerebellum		-15	-84	-45	2.47
L	Middle temporal gyrus	88	-54	-15	-21	2.91
	Temporal pole		-57	3	-21	2.65
	Middle temporal gyrus		-45	-12	-15	2.53
L	Middle temporal gyrus	88	-63	-57	6	2.64
	Inferior temporal gyrus		-54	-63	-18	2.59
	Lateral occipital cortex		-57	-66	-3	2.2
**Increased Connectivity**						
R	Superior parietal lobule	76	24	-36	42	3.37

Significant (*n*=280, $p<0.05$, FWE-corrected) clusters, identified at $p<0.001$, uncorrected, ordered by cluster size K_E (each voxel=27 mm^3). For completeness, we also report the clusters' peak voxels and their T-scores. L=left hemisphere; R=right hemisphere

SUPPLEMENTARY NOTE 1 | Accounting for differences in demographics. To account for a possible influence of additional variables on our main behavioural results, we conducted regression analyses that factored out the main covariates of no interest. These included gender, level of education (both entered as categorical variables as in Table 1) and handedness (entered as the Edinburgh handedness score [61]), as well their interactions with age. The covariate of interest was age, and we ran three models with: 1) Direct force overcompensation; 2) Direct intercept; and 3) Slider slope as the dependent variables. All variables were z-score scaled before being entered into the model.

We found that neither of the covariates of no interest, nor their interactions with age, were significant predictors of Direct force overcompensation (n=322; all p>0.075), Direct intercept (n=322; all p>0.065) or Slider slope (n=322; all p>0.11). Importantly, however, in these analyses, age remained a significant predictor of Direct overcompensation (n=322, beta=0.25, p=1.07e-05), Direct intercept (n=322, beta=0.25, p=6.99e-06) and Slider slope (n=322, beta=-0.27, p=1.15e-06). These results suggest that gender, education and handedness did not significantly influence the main behavioural results reported in the study.

SUPPLEMENTARY NOTE 2 | Matched forces across different time windows.

We tested whether the time window used to assess sensory attenuation influenced our results, for example due to differences in the time course of response with age. To this end, we measured the matched force over two other 0.5 s windows, starting at 2.5 and 3.0 s, into the matching period, compared to the standard window starting at 2.0 s. The matched forces in the standard window correlated strongly with the later time windows (2.5 s and 3 s) in both the Direct (r=0.997 and 0.989, respectively) and Slider conditions (r=0.998; 0.993). Moreover there were no correlations between age and the differences in matched forces between the standard window and each of the two other windows in the Direct (n=322, r=0.018 and 0.028; both p>0.6) and Slider condition (n=322, r=0.101 and -0.071; both p>0.05). Together, these results suggest that the differences between the matched forces calculated from the standard time window and the later windows did not vary as a function of age. We also re-ran the main correlation analyses on the two supplementary time windows for both conditions (Supplementary Table 1). The results of these analyses were very similar to the results of the standard time window.

SUPPLEMENTARY NOTE 3 | Variability in Slider force overcompensation. In addition to assessing sensory pressure sensitivity from the slope of the linear regression of the target and matched forces, we also assessed variability in pressure perception in the Slider condition. We examined the variability in Slider force overcompensation (i.e. matching errors) as an alternative measure for sensory differences with age, for estimating the precision of sensory signals. This is perhaps a more direct measure of each participant's sensory precision and it also does not assume a linear relation (as does the slope). However, overcompensation variability is more susceptible to outlier trials.

We examined the standard deviation of force overcompensation for each participant, after excluding one trial in which overcompensation was farthest from the mean to reduce the influence of outlier trials. There was a significant correlation between age and each participant's standard deviation across participants (n=322, Spearman's rho=0.18, p=0.002), suggesting increased variability in the perception of forces with ageing.

As found with the slope (see main text), variability in overcompensation in the Slider condition was correlated with Direct intercept (n=322, Spearman's rho=0.22, $p<0.001$), over and above the effect of age (partial correlation of Direct intercept and Slider variability with age factored out; n=322, Spearman's rho=0.19, $p<0.001$). Finally, factoring out Slider variability weakened the relation between Direct intercept and age by 12% (proportion of Spearman's rho reduced when including Slider variability in a partial correlation). The shared variance of Slider variability explained 32% of the total variance of the linear relation of Direct intercept and age.

Together, the pattern of results that emerged when using perceptual variability in the Slider condition as a measure that reflects sensory precision was similar to that reported in the main text for the slope. Both these measures suggest reduced sensory precision with age, in agreement with the extensive literature showing a sensory decline with age.

SUPPLEMENTARY NOTE 4 | Generic overcompensation process. Although we interpret the main behavioural results as an age-related increase in the reliance on sensorimotor predictions, alternative interpretations must also be considered. Mainly, it is important to consider the effects of a general overcompensation process (discussed here) and sensory deprivation (discussed in Supplementary Note 5).

Older adults could apply larger forces as a generic compensatory response. For example, older adults could use a cognitive overcompensation strategy because they cannot sense the pressure. Such a cognitive strategy in sensory impaired participants, whether conscious or subconscious, could in principle lead to increased force overcompensation with ageing. Importantly, in the case of an overcompensation cognitive strategy, one would also expect additional effects on the results as follows:

1) A cognitive overcompensation strategy is unlikely to be specific to the Direct condition, as participants are presented with the same level of forces in both the Direct and Slider conditions. Hence, an increased overcompensation should also emerge in the Slider condition. However, in the Slider condition there was reduced force overcompensation as a function of age, suggesting older adults were overall more accurate. It is not readily clear how a cognitive overcompensation strategy would be specific to the Direct condition only.

2) The overcompensation in sensory deprived participants would be specific or at least more prominent for the smaller target forces, which are more difficult to sense. Therefore, overcompensation should be larger for the smaller force levels and relatively smaller for the larger force levels, since larger force levels are more clearly

perceived by sensory deprived participants. This behavioural pattern would be expressed in a flattening of the matched-target force regression slope. Interestingly, this age-related flattening of slope was observed in the Slider condition, but critically, it was not observed in the Direct condition, where the slope does not change with age. In other words, in the Direct condition, increased overcompensation was observed consistently for all force levels.

In summary, while a general overcompensation strategy (for example a cognitive strategy due to sensory deficits) cannot easily explain all the behavioural results, we raise this as an additional potential caveat.

SUPPLEMENTARY NOTE 5 | Sensory deprivation in older adults. Sensory deprivation (e.g. by neuropathy) is common in normal ageing, which can influence the performance in many perceptual paradigms. By using the Force Matching Task, we minimise the impact of such changes, as consistent biases, such as those resulting from sensory deprivation, are factored out. Therefore, as the task requires the matching of presented (target) forces, participants can still be accurate in the task despite having sensory deficits (reduced sensory gain or thresholds). For example, if presented with a 2 N force but perceiving it at only 50% (i.e. 1 N), participants would still have to apply a 2 N force in order to match the perceived force (of 1 N), in either Direct or Slider conditions.

There are, however, specific scenarios in which sensory deprivation could nonetheless influence the matching paradigm. In the task, participants are instructed to attend and perceive the force applied to their left index finger. It is possible that when matching the force in the Direct condition, they used their right finger for sensing the applied force. By itself this should not influence the behavioural results in the case of a bilateral, symmetric sensory deprivation (as it would lead to the same result as in the example above). However, it may yet influence the results if participants have an asymmetric sensory deficit, which specifically affects their right finger more strongly than their left finger. Then, for example, if participants are presented with a 2 N force which they perceive accurately with their left finger, they will have to apply a larger force in order to experience 2 N with their impaired right finger. Although this cannot be ruled out, it is unlikely that this combination of altered task strategy and specific right hand sensory deficit would be consistent enough to drive the behavioural effects we observe in a large population-based cohort. Moreover, it is unclear how this

interpretation can explain the distinct effects of age on behaviour in the Direct condition and in the Slider condition. Still, we raise this as a potential caveat for our study.

SUPPLEMENTARY METHODS | Univariate analysis of fMRI movement task.

Task-related fMRI analysis was performed to establish the relation between sensorimotor attenuation and BOLD signal invoked by the sensorimotor task in the scanner. Preprocessing and first-level analysis were performed as part of the pipeline of the Cambridge Centre for Ageing and Neuroscience (Cam-CAN) data repository [33]. In brief, normalised and smoothed images (see main Methods section for more details on preprocessing) were fitted with a general linear model after modelling the BOLD signal with a haemodynamic response function. Regressors included stimulus (multi- and unimodal events separately) and null event onsets, as well as six motion parameters (head motion and harmonic regressors as in Tsvetanov et al. [32]). The fMRI data for the same 280 participants whose data were analysed for the functional connectivity were included. The event onsets for two participants could not be retrieved due to a technical error, making up a total of 278 participants. Second-level analysis was performed on the first-level contrast: multimodal stimuli against null events. A multiple regression analysis was run with a similar set of regressors to that of the functional connectivity analysis in the main text, including: age, coil (before versus after coil change), handedness, gender and total motion. Although there was no correlation between age and mean reaction time (n=278, Spearman's rho=-0.034, p=0.569), mean reaction time was also included in the general linear model as a covariate of no interest for both task-related univariate analysis and functional connectivity analysis. The main result reported was the t-map for the mean effect of stimuli (positive contrast of the constant term in the general linear model).
